# Supplementary material for: The Study of Metschnikowia pulcherrima E1 in the Induction of Improved Gray Spot Disease Resistance in Loquat Fruit
Source: J Fungi (Basel). 2025 Jun 30;11(7):497. doi: 10.3390/jof11070497 (PMC12298231; doi:10.3390/jof11070497)
Supplement: Supplementary file 1 [file jof-11-00497-s001.zip › jof-3688627-supplementary.pdf]

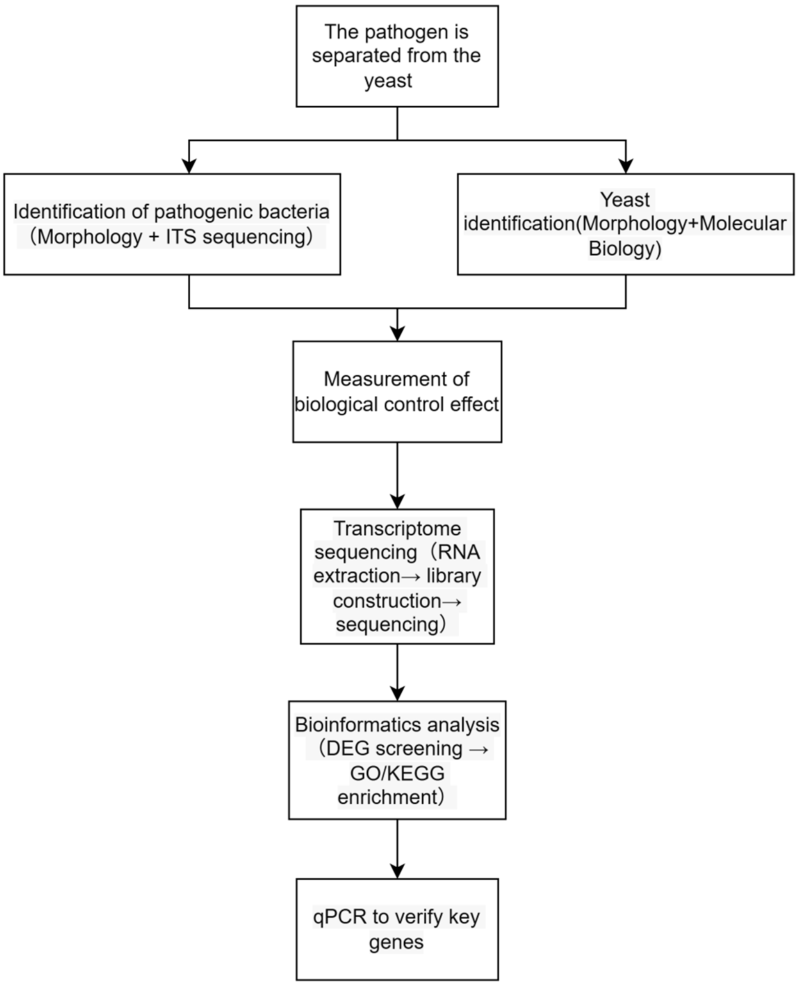

Figure S1. Experimental flow chart.

Table S1. Primer design of differentially expressed genes.

Gene ID	Gene Name	Revers Primer (5′ to 3′)
1	*CML19*	F: ACACAAGAGCACTGCAACTG<br>R: TACACCCAGCAGCTTGAAGA
2	*FLS*	F: GTGGGAGGTGAAGAAGTGGA<br>R: TAGTCGACAGCAACCCAGTT
3	*ACT*	F: CTGCTAGCGAACTCCCCATA<br>R: GATCCCGCTCCTATGTTGGT
4	*CML42*	F: TGGCCTCTACAGTTTCAGGG<br>R: TATCGTGGAATGGAGGTCGG
5	*CYP749A22*	F: ACACAAGAGCACTGCAACTG<br>R: TACACCCAGCAGCTTGAAGA
6	*XTH23*	F: TATCCTCATCGGCTGGCTTT<br>R: GGCAACAGAGAGCAGCAATT
7	*AFS1*	F: AGGCCTAGTTTTCGGAAGCT<br>R: CCCCAACCTGAAGCCTCTTA
8	*CP1*	F: CAACGGCCAGGATCTTAAGC<br>R: AAAAGCTCGGGAGTGATGGA
9	*HST*	F: CCGCCTCAACAAACAGAACA<br>R: CTACAGGCCGAATCCAAACG
10	*FHY*	F: CCTTGCGGATACTGTGCTTC<br>R: ACAGCCCAACCGAAGAAAAC
11	*GID1C*	F: CCTTGCGGATACTGTGCTTC<br>R: ACAGCCCAACCGAAGAAAAC
12	*Actin*	F: ACCATCCAGCATGTAACCCA<br>R: TGTGGCCTGCTCAAGATACA
